# Review: Retinal degeneration: Focus on the unfolded protein response

**Published:** 2013-09-20

**Authors:** Marina Gorbatyuk, Oleg Gorbatyuk

**Affiliations:** 1 Department of Vision Sciences, University of Alabama at Birmingham, Birmingham, AL 35233,; 2 Department of Molecular Genetics and Microbiology, University of Florida, Gainesville, FL 32610

## Abstract

Recently published literature has provided evidence that the unfolded protein response (UPR) is involved in the development of retinal degeneration. The scope of these studies encompassed diabetic retinopathy, retinopathy of prematurity, glaucoma, retinal detachment, light-induced retinal degeneration, age-related macular degeneration, and inherited retinal degeneration. Subsequent studies investigating the role of individual UPR markers in retinal pathogenesis and examining the therapeutic potential of reprogramming the UPR as a method for modulating the rate of retinal degeneration have been initiated. Manipulation of UPR markers has been made possible by the use of knockout mice, pharmacological agents, and viral vector-mediated augmentation of gene expression. Future research will aim at identifying specific inhibitors and/or inducers of UPR regulatory markers as well as expand the list of UPR-related animal models. Additionally, adeno-associated virus-mediated gene delivery is a safe and effective method for modulating gene expression, and thus is a useful research tool for manipulating individual UPR markers in affected retinas and a promising delivery vector for gene therapy in retinal degenerative disorders.

Retinal degeneration is progressive deterioration of the retinal cells, eventually culminating in their death. Certain conditions can lead to an imbalance in the retinal microenviroment, which in turn could cause retinal degeneration. These conditions include but are not limited to artery or vein occlusion in diabetic retinopathy, hypoxic retina in retinopathy of prematurity, aging in age-related macular degeneration, expression of mutant proteins in inherited retinal degeneration, traumatic injury leading to retinal detachment, and light sensitivity in the case of light-induced retinal degeneration. Despite recent advances in our understanding of the mechanism of these retinopathies, much work remains to be done to get a clear picture of the molecular pathology of each disease. The understanding of the underlying mechanisms is complicated by the heterogeneity of cases within a particular retinopathy and by the interplay of multiple cellular signaling involved in each disease model.

The unfolded protein response (UPR) or the endoplasmic reticulum (ER) stress response is a series of evolutionarily conserved signaling pathways aimed at restoring homeostasis under conditions of ER stress [1]. ER homeostasis can be compromised by various stimuli, including disturbances in redox regulation [2], calcium regulation [3,4], glucose deprivation [5,6], and viral infection [7,8]. During ER stress, the accumulation of unfolded proteins in the ER lumen activates pancreatic ER kinase (PKR)-like ER kinase (PERK) [9,10], inositol-requiring kinase/endoRNase 1 (IRE1) [11], and activating transcription factor 6 (ATF6) [12] proteins and initiates the three associated UPR signaling pathways (Table 1, Figure 1A). The molecular chaperone glucose-regulated protein 78 (*GRP78*) or binding immunoglobulin protein (BiP), in addition to Ca^2+^ binding and protein processing functions, possesses one more key role: master initiator of early UPR signaling. In resting conditions, GRP78 is bound to these mediators. The association of GRP78 with the ER luminal domains of these mediators prevents their activation and maintains the UPR signaling machinery in an inactive state [11]. Accumulation of unfolded proteins in the ER lumen triggers the dissociation of GRP78 from its quiescent UPR mediators. This dissociation from PERK, ATF6, and IRE1 satisfies the demand for appropriate protein folding. The free and active PERK, ATF6, and IRE1 then orchestrate a series of transformations known cumulatively as the UPR [13].

**Table 1:** List of UPR markers.

PERK	Pancreatic ER kinase (PKR)-like ER kinase	PERK signaling. Once activated phosphorylates eIf2α and blocks general protein synthesis.
IRE1	Inositol-requiring kinase/endoRNase 1	IRE1 signaling. Once activated IRE1 removes a 26-nucleotide intron from the XBP1 mRNA. May activate the c-Jun N-terminal kinase (JNK) pathway.
ATF6	Activating transcription factor 6	ATF6 signaling. Once activated translocates to the Golgi where it is cleaved by proteases. Active ATF6 translocates to the nucleus and induces genes with ER stress response element (ERSE) such as GRP78. GRP94, CHOP, XBP1.
GRP78 or Bip	Glucose-regulated protein 78 or binding immunoglobulin protein	Key player of PERK, ATF6, IRE1 signaling. On accumulation of unfolded proteins dissociates from the three receptors PERK, ATF6 and IRE1 leading to their activation and triggers the UPR.
eIF2α	Eukaryotic transcription factor 2α	PERK signaling. Phosphorylation of eIF2α by activated PERK blocks protein synthesis by attenuating of capor-eIF2α-dependent translation.
ATF4	Activating transcription factor 4	PERK signaling. Carries internal ribosomal entry site (IRES) in five prime untranslated region and escapes the eIF2α-dependent translation block. Induces expression of genes involved in amino acid metabolism, redox reactions, stress response, protein secretion and pro-apoptotic CHOP protein.
CHOP	C/EBP homologous protein or GADD153 (Growth Arrest and DNA Damage inducible gene 153)	PERK, ATF6, IRE1 signaling. Pro-apoptotic protein. Important element of the switch from pro-survival to pro-death signaling. Apoptosis-induced targeted genes are Bcl2 (downregulation), GADD34, TRB3, Ero1.
GADD34	Growth Arrest and DNA Damage inducible gene 34. a protein phosphatase 1 (PP1)-interacting protein	PERK signaling. Causes PP1 to dephosphorylate eIF2α and thus release the translational block. Expression of GADD34 correlates with apoptosis.
XBP1	X box-binding protein 1, transcription factor	IRE1 signaling. Undergone by splicing (26bp) to be active. Translocates to the nucleus and controls the transcription of chaperones
P58IPK	Interferon-induced protein kinase PKR, Hsp40 family member (DnaJC3)	IRE1 signaling. Binds and inhibits PERK providing negative feedback loop and relieves the PERK-mediated translation block.

**Figure 1:**
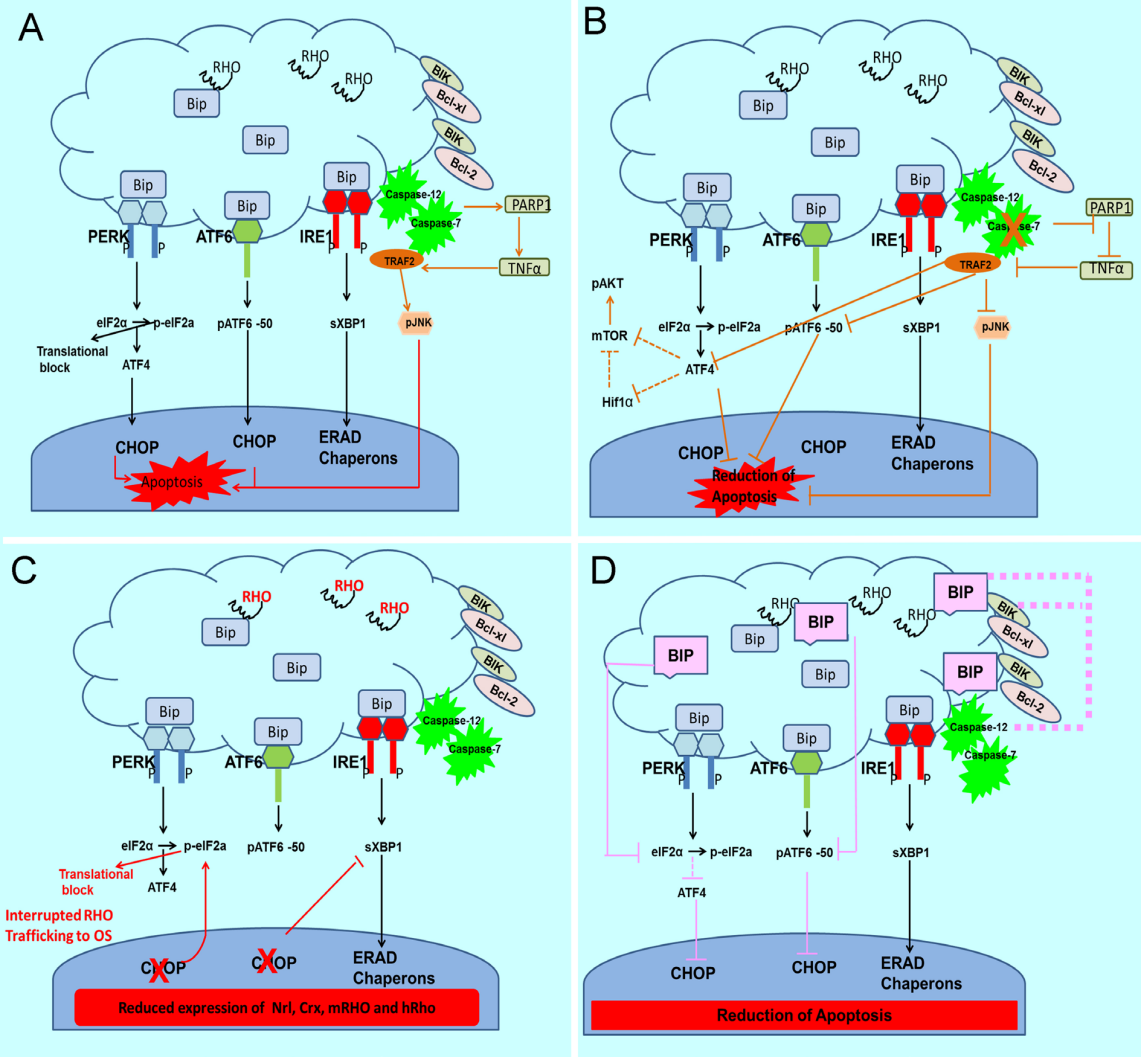
The unfolded protein response (UPR) is activated in the autosomal dominant retinitis pigmentosa (adRP) retina and proposed to be a target for modulating the rate of retinal degeneration. **A**: Activation of the UPR occurs in the adRP retina [65,67,72]. The endoplasmic reticulum (ER) stress mediators pancreatic ER kinase (PKR)-like ER kinase (PERK), inositol-requiring protein 1 (IRE1), and activating transcription factor 6 (ATF6) are activated during ER stress. During normal cellular homeostasis, the immunoglobulin heavy chain-binding protein (GRP78/BiP) is tightly bound to all three mediators. In the presence of unfolded proteins, BiP dissociates from the three mediators to assist in appropriate protein folding leading to activation of the PERK, ATF6 and IRE1 UPR arms. In addition to endoRNase activity, phosphorylated (p) IRE1 can activate the c-Jun N-terminal kinase (JNK) that is known to induce apoptosis through tumor-necrosis factor (TNF)-receptor-associated factor 2 (TRAF2) and ASK1 (apoptotic signaling kinase 1) [25,91]. In mammals, recruitment of TRAF2 by pIRE1 allows it to signal to c-Jun N-terminal kinase. The IRE1–TRAF2 complex has also been linked to caspase-12 activation and cell death. Caspase-12 is activated [92] in addition to the calpains and Ca2+-induced activation by caspase-7 that translocates to the ER during ER stress [93]. Activation of the UPR in the adRP retina is accompanied by upregulation of the TNFα and poly (ADP-ribose) polymerase 1 (PARP1) proteins [72,94]. **B**: CASP7-ablation in the T17M *RHO* retina leads to a decreased rate of retinal degeneration in T17M *RHO* mice. We found that ablation of CASP-7 in adRP retinas leads to a therapeutic effect detected by electroretinogram (ERG), optical coherence tomography (OCT), and histology [72]. The CASP-7 ablation also modulates UPR signaling. Reduction in BiP, ATF6, ATF4, Bim, Bik, Edem2, and eIF2α was detected, suggesting that UPR signaling is modulated in these mice. The second pathway modified by ablation of CASP-7 is the mammalian target of rapamycin/protein kinase B (mTOR/AKT) signaling resulting in downregulation of mTOR and increase of pAKT in T17M *RHO* CASP-7 mice. Finally, ablation of CASP-7 led to the diminishing of TRAF2-JNK-induced apoptosis. This reduction may have occurred through the inhibition of PARP1, which is known to be cleaved by CASP-7. The PARP1 inhibition in turn perhaps led to the downregulation of TNFα resulting in a decrease in TRAF2-pJNK signaling through PARP1-TNFα-tumor necrosis factor receptor type 1-associated death domain-receptor-interacting serine/threonine protein kinase 1-(TADD-RIPK1)-ASK1-pc-JUN. **C**: Ablation of proapoptotic CHOP accelerated retinal degeneration in adRP mice through reprogramming the UPR. Detrimental effects from the ablation of proapoptotic CHOP were detected with ERG, OCT, and histology [71]. We found that ablation of CHOP leads to a greater than eightfold increase in peIF2α and 30% reduction in spliced Xbp1. These changes were accompanied by accumulation of the RHO protein in the cytoplasm of the photoreceptors [71]. Therefore, we concluded that ablation of CHOP in adRP photoreceptors may be linked to global inhibition of protein translation. An observed increase in the histone deacetylase 1 (HDAC1) levels supported this hypothesis. **D**: Overexpression of GRP78/BiP in the adRP retina slowed the rate of retinal degeneration. Adeno-associated viral (AAV) delivery of GRPP78/BiP to adRP photoreceptors led to an increase in ERG amplitudes during 3 months [65]. Overexpression of BiP reprograms the UPR by reducing peIF2α (the PERK pathway) and pATF6-50 (the ATF6 pathway) leading to a decrease in proapoptotic CHOP. In addition, BiP binds to caspase-12, thus further preventing from induction of apoptosis. Finally, BiP forms a complex with Bcl-interacting killer (BIK) preventing the translocation of Bcl-2-associated X protein (BAX) and Bcl-2 homologous antagonist killer (BAK) to the mitochondria. We thus concluded that preservation of photoreceptor function resulting from elevated levels of BiP is due to suppression of apoptosis rather than to promotion of rhodopsin folding.

Increased expression of GRP78 may serve as an indicator of UPR activation [14,15]. However, once the need for the chaperone activity of GRP78 is satisfied, it gradually reassociates with PERK, IRE1, and ATF6, thus inactivating these signaling mediators, resolving the UPR signaling, and reestablishing homeostasis.

ER stress leads to the upregulation of PERK. The initial phase of UPR activation consists of translational attenuation and cell cycle arrest mediated by PERK. Dissociation of BiP from PERK initiates dimerization and autophosphorylation of the PERK kinase domain [16]. Activated PERK reduces global protein production by inducing the phosphorylation (p) of eukaryotic transcription factor 2α (eIF2α) [17]. Phosphorylation of eIF2α at Ser 51 interferes with the formation of an active 43S translation-inhibition complex and suppresses the translation [18]. At the same time, peIF2α allows translation of messenger RNAs (mRNAs) with short open reading frames in their 5′-untranslated regions, including activating transcriptional factor 4 (ATF4) [19]. ATF4 in turn controls the expression of important genes involved in apoptosis such as C/EBP-homologous protein (CHOP) and growth arrest and DNA damage-inducible 34 (GADD34) [19] (Table 1). The latter participates in a feedback loop that controls phosphorylation of eIF2α and, thus, participates in restoring protein synthesis [19].

ATF6 is best known as the cyclic adenosine monophosphate response element (CRE)/ATF transcriptional factor that regulates expression of multiple genes associated with the UPR [20]. This 90-kDa protein has a basic leucine zipper domain (bZip) for DNA binding post homo- or heterodimerization. ATF6 binds to the ER stress response elements (ERSEs), UPR element (UPRE), and ERSE-II in a promoter [21] to transmit stress signals directly from the ER to the nucleus [20]. Under ER stress conditions, ATF6 dissociates from BiP and is transported on vesicles toward the Golgi apparatus, where ATF6 undergoes processing by first the site-1 and then the site-2 proteases. The N-terminal fragment of pATF6 (cleaved form) translocates to the nucleus and promotes transcription of UPR genes, such as BiP and the transcriptional factors CHOP and X-box binding protein 1, also known as XBP1. It also promotes transcription of other proteins such as p58IPK, best known as an inhibitor of the eIF2α protein kinases PKR and PERK [22], and sarco/endoplasmic reticulum Ca^2+^-ATPase (SERCA) [20].

IRE1 represents the most highly conserved branch of the UPR. IRE1’s C-terminal cytosolic effector region manifests kinase and endoRNase activity [1]. IRE1 senses unfolded proteins in the ER lumen by activating the cytoplasmic kinase and endoRNase domains [23]. The activation of IRE1 is accompanied by oligomerization and autophosphorylation within the cytosolic effector region [20]. Under ER stress conditions, IRE1 catalyzes unconventional splicing of Xbp1 mRNA, which generates a protein that differs from its unspliced variant (uXbp1). Xbp1 is a potent transactivator that regulates genes involved in ER protein synthesis, folding, glycosylation, ER-associated degradation, redox metabolism, autophagy, and vesicular trafficking [1]. IRE1 also activates c-Jun NH2-terminal kinase (JNK) by recruiting the tumor necrosis factor (TNF) receptor-associated factor 2 (TRAF2) [24,25] in the presence of mitogen-activated protein kinase kinase kinase (MAP3K), apoptosis signal-regulating kinase 1 (ASK1), and ASK1-interacting protein-1 (AIPI-1). This activation is associated with cell death [20,26]. IRE1 signaling is attenuated after prolonged ER stress. This process is characterized by IRE1 dephosphorylation and a decrease in endoRNase activity [20].

The primary goal of the UPR is to reestablish homeostasis while maintaining a prosurvival signaling environment. However, if the ER capacity to deal with persistent stress is insufficient, the UPR-associated signaling shifts from a prosurvival to a proapoptotic cascade that eventually becomes dominant and leads to cell death. This shift can result in the accumulation of proapoptotic CHOP and IRE-regulated JNK apoptosis [27,28]. This dual nature of UPR-associated signaling has in fact been likened to the “yin” and “yang” with the prosurvival GRP78 (yang) and proapoptotic CHOP (yin) signaling comprising opposing components of the same larger cellular mechanism [13].

## The unfolded protein response and retinal degenerative diseases

The ER stress response has been recently proposed as a contributing factor to retinal degenerative disease [29,30]. The fact that UPR activation can induce retinal cell death in mice has been previously reported [31]. Despite the physiologic responses from retinas treated with tunicamycin or N-methyl-D-aspartic acid (NMDA) or retinas with elevated intraocular pressure (IOP) that had not been assessed with electroretinogram (ERG), these authors clearly demonstrated that the activation of ER stress by itself could provoke retinal degeneration. In real-world conditions, activation of the ER stress response often occurs in retinas with other preexisting complications, and little is known about the cellular consequences of the UPR upon physiologic levels of ER stress in vivo [29].

For the past decade, our attention has been drawn to the role of the UPR in retinal degenerative diseases. The majority of the studies on retinal degenerative diseases identified the UPR as a common cellular pathway involved in retinal diseases but were unable to ascertain its exact role in the disease process. Although there have been valuable insights into the interplay of the UPR and retinal pathology, it remains unclear whether the UPR is directly responsible for causing retinal degeneration or alternately if UPR activation is in response to retinal cell death. This key question remains to be answered by future research. With that said, in this review we will summarize the active status of research regarding the UPR in retinal degenerative diseases and will highlight progress that has been recently made in our understanding of UPR’s precise role.

## Diabetic retinopathy

Recently, the Eye Disease Prevalence Research Group reported that “one in every 12 individuals with diabetes age 40 years and over has vision-threatening diabetic retinopathy.” This frightening number points to the importance of investigating the mechanism of diabetic retinopathy (DR), which remains unresolved. In the case of DR, the cellular signaling pathways leading to vision impairment and retinal neovascularization are of particular interest.

A study demonstrating UPR involvement in the cellular mechanism of DR was conducted in 2006 by Ikesugi et al. [32]. The authors demonstrated that glucose fluctuation, in particular hypoglycemia and not hyperglycemia, activated UPR-specific enzymes in pericytes, cells that participate in the proliferation of DR. These studies were conducted in cultured pericytes. However, subsequent work in diabetic Akita mouse [33] and rat retinas [34] demonstrated the activation of the ER stress response, suggesting that it may be one of the earlier events in DR progression.

## Retinopathy of prematurity

A study conducted with the mouse model of oxygen-induced retinopathy (OIR) demonstrated that GRP78 is significantly upregulated in the OIR retina at the RNA and protein levels. In addition, the expression of ATF4 and the amount of phosphorylated eIF2α were also significantly upregulated suggesting that ER stress is enhanced in the OIR retina [33]. Recently, Nakamura et al. [35] also established a link between ER stress and the pathological neovascularization in the DR retina and demonstrated that the triggering of ER stress by injection of tunicamycin or thapsigargin in mouse OIR retinas accelerated retinal neovascularization. These data point to the UPR as a cellular signaling cascade that could be reprogrammed to reduce neovascularization and halt the proliferation of DR.

## Glaucoma

Glaucoma is a term for a heterogeneous group of ocular neuropathies leading to gradual axonal degeneration in the optic nerve, progressive loss of retinal ganglion cells, visual impairment, and irreversible blindness. It has been estimated that 80 million people will be affected by this disease in 2020 worldwide [36]. Emerging evidence indicates that increased IOP, decreased neutrophine supply [37,38], hypoxia [39], excitotoxicity [40,41], oxidative stress [42], and autoimmune [43-45] processes all interact as pathogenic markers and may collectively be responsible for the development and progression of glaucoma.

Recently, transgenic mice expressing the mutant myocilin (MYOC) (Y437) mimicking human phenotypes seen in patients with primary angle glaucoma (POAG) have been developed [46]. These mice have been used to study the mechanism of POAG. The results have demonstrated that chronic and persistent ER stress is associated with trabecular meshwork cell death and elevation of IOP in Tg-MYOC(Y437H) mice. The authors proposed that ER stress in these mice is linked to the pathogenesis of POAG and may be a therapeutic target in human patients [46].

## Retinal detachment

Retinal detachment (RD) is a common cause of human visual impairment and can be provoked by microenvironmental events such as the presence of one or more small holes or tears in the retina or traumatic injury, such as that sustained in an accident or in combat. This disorder is characterized by the peeling of the retina away from its underlying layer of support tissue. This separates the retina from the back of the eye and causes the retina to detach. The detached portion of the retina does not function properly resulting in a blur or a blind spot in the visual field. Progression of retinal detachment results in an increasing blurring and loss of the visual field with significant or total loss of vision likely unless the detachment is repaired.

A study conducted with a rat model of retinal detachment demonstrated that GRP78 and CHOP/GADD153 mRNA and protein levels were elevated in retinas in a time-dependent manner [47]. Elevated expression of GRP78 was observed in all layers of the retina in rats with retinal detachment while expression of the CHOP was most prominent in photoreceptors. The authors therefore concluded that the ER stress-related markers were upregulated in response to retinal detachment.

Another study conducted in the rat model of RD also confirmed previous results by detecting the proapoptotic CHOP mRNA and protein levels as they were upregulated from day 1 to day 7 post-detachment [48]. These studies also suggested that ER stress–mediated apoptosis is involved in retinal detachment-induced photoreceptor cell death.

## Light-induced retinal degeneration

The role of ER stress in light-induced retinal degeneration has recently been verified. In 2008, Yang et al. [49] found that after light exposure, the ER stress markers GRP78/BiP, caspase-12, peIF2 alpha, and PERK were significantly upregulated in a time-dependent manner. Interestingly, the authors observed that the upregulation of these proteins coincided with apoptosis of the photoreceptors. Based on these observations, the authors proposed that ER stress plays an important role in light-induced photoreceptor apoptosis and that, consequently, ER stress modulators could be strong therapeutic candidates for treating retinal degeneration. We have also demonstrated that BiP and CHOP expression is elevated in the retina of BALB/c mice exposed to constant light and peaks at 4 h after light exposure [50]. Furthermore, a study of 661W cells, a mouse retinal photoreceptor-derived cell line, exposed to light and mice with light-induced retinal degeneration demonstrated that light exposure induces upregulation of polyubiquitinated proteins, S-opsin aggregation, and an increase in BiP and CHOP mRNA levels and cell death [51]. This study used 661W cells as an effective in vitro model for the photoreceptor cell response to visible light since these cultured cells demonstrate light-induced cell death in a manner similar to that observed in vivo [52,53]. Given these findings and our own observations, we believe that the manipulation of ER stress markers may also be helpful in treating this environmental form of retinal degeneration.

## Age-related macular degeneration

Age-related macular degeneration (AMD) is a multifactorial disease affecting an estimated 10 million Americans and 50 million people worldwide with several risk factors, including aging, genetic predisposition, smoking, obesity, and hypertension [54,55]. Recently, animal models of AMD, which manifest some of the features of the human disease, have become available [56]. However, due to the complexity and progressive characteristics of AMD, the majority of these models do not recapitulate the full spectrum of the human AMD pathology.

For example, ER stress could be an important mechanism in the pathogenesis of AMD and, with oxidative stress, could trigger inflammation and disease [57]. However, another study suggested that in addition to oxidative stress, impaired phagocytosis, elevation of polyunsaturated fatty acids, and exposure to light can provide ideal conditions for the development of retinal degeneration in the macular region. That said, in human retinal pigment epithelium (RPE) cells, oxidative stress leads to inhibition of proteosomal activity and aggresomal accumulation of ubiquitinated proteins [58,59], thus suggesting that prolonged oxidative stress is in turn capable of activating the ER stress response.

Yoshikawa et al. aimed at determining how ER stress affects the expression of tight junction molecules in RPE cells [60]. To do this, they exposed cultured ARPE-19 cells, a human RPE cell line, with the ER stress inducers tunicamycin and thapsigargin. This study also revealed that induction of ER stress in ARPE-19 cells not only led to elevated gene expression from the UPR and vascular endothelial growth factor (VEGF) but also provoked increased expression of tight junction proteins, thus altering the function of RPE cells. This alteration of protein expression and cellular function may be yet another way in which the ER stress response could be involved in the pathogenesis of AMD.

The proposed effects of light-induced damage of RPE and oxidative stress on AMD were recently tested in albino rats [61]. The levels of spliced Xbp1 and GRP78 increased in the retina but were remarkably suppressed in the RPE. This suppression correlated with an increase in the antioxidant genes nuclear factor (erythroid-derived 2)-like 2, superoxide dismutase 2, and nitrotyrosine 3, which are markers of oxidative stress. These findings suggested that the disruption of Xbp1 activation may be linked to decreased antioxidant gene expression and increased oxidative stress during light-induced damage in the RPE, thus leading to RPE pathogenesis.

## Inherited retinal degeneration

Involvement of the UPR in the mechanism of inherited retinal degeneration has been studied in vitro and in vivo. In a study of inherited early-onset macular degenerative disease, ARPE-19 cells were used in investigating the mechanisms of malattia leventinese and Doyne honeycomb retinal dystrophy [62], which previously were thought to be linked to the R345W mutant of fibulin-3. That same study found that overexpression of R345W fibulin-3 caused activation of the UPR and increased VEGF expression when compared with overexpression of the wild-type protein. This suggested that the ER stress response leads to RPE dysfunction and contributes to the pathogenesis of these disorders.

Subsequent availability of animal and *Drosophila* models of retinitis pigmentosa (RP) has allowed scientists to extensively study the UPR in the degenerating retina. For example, in the *Drosophila* retina expressing the ninaE^G69D^ protein, a homolog of mammalian opsin, leading to an autosomal dominant RP (adRP), splicing of Xbp1 (the IRE pathway), and induction of hsc3 (*Drosophila* homolog of GRP78/BiP) have been demonstrated [63]. Another study of *Drosophila* expressing the Rh1P37H, a homolog of mammalian P23H rhodopsin (RHO) demonstrated that the activation of Ire/Xbp1 signaling is associated with the suppression of photoreceptor neuron degeneration and blindness when the valosin containing protein, an ER-associated degradation effector and binding partner of Rh1P37H, was genetically inactivated [64]. This study suggested activated Ire1 signaling was associated with a therapeutic effect on the fly’s retina.

We have also observed the activation of this signaling pathway in adRP transgenic rat retinas expressing the P23H *RHO* [65], S334ter *RHO* [66], and mouse retinas expressing T17M *RHO* [67]. This splicing has been observed in parallel with activation of PERK and ATF6 (P23H and S334ter RHO rats) signaling and has confirmed that ER stress contributes to the mechanism of adRP.

Activation of the UPR has also been shown in another adRP model produced by the R69H and R129S mutants of carbonic anhydrase IV [68]. Using a transient expression system in COS-7 cells, the authors found that the underlying retinal molecular pathogenesis was associated with impaired protein trafficking to the cell surface and with activation of ER stress markers. In addition, the expression of both CA IV mutants induced elevated levels of BiP and CHOP associated with apoptotic cell death.

In addition to adRP, the cellular signaling of autosomal recessive RP (ARRP) retina has also been investigated. Progression of ARRP is closely associated with the UPR [69]. The retinas of rd1 mice carrying the nonsense mutation within rod cyclic guanosine monophosphate phosphodiesterase β subunit express the hallmarks of the activated UPR such as increased protein levels of BiP, caspase-12, and peIF2α in a time-dependent manner. These changes were accompanied by photoreceptor apoptosis suggesting that the ER stress contributed to retinal pathogenesis in rd1 mice.

In other forms of inherited retinal degeneration such as achromatopsia, progressive cone dystrophy, and AMD previously linked to the R563H and Q655X mutations of the cyclic nucleotide gated channel alpha 3 (CNGA3) subunits, activation of the UPR has also been demonstrated [70]. In 661W cells, these proteins display altered degradation rates and are retained in the ER. This ER retention was associated with increased expression of the UPR-related markers pPERK, BiP, and CHOP and with decreased cell viability. These results led to the proposal that ER stress can arise from expression of localization-defective CNG channels, and may represent a contributing factor to photoreceptor degeneration.

The most recent studies have linked ER stress to retinal degeneration, raising the question of whether ER stress is protective or proapoptotic. Recent work in mice [61,71] and flies [29] has demonstrated that the UPR may be beneficial to retinal cells during retinal degeneration and that the activation of UPR markers could be a part of retinal cellular defense. Mendes et al. proposed that mild ER stress protects photoreceptor neurons from various death stimuli in adult *Drosophila* [29]. Another study with animal models of retinal degeneration suggested that therapies aimed at reduction of ER stress or upregulation of ER stress-induced protective molecules in the retina could be beneficial for preserving vision [62].

## Role of individual unfolded protein response markers in the mechanism of retinal degeneration

A great deal of literature suggests that UPR signaling could be targeted for therapeutic intervention in the degeneration of impaired retinal neurons. Reprogramming of the UPR could slow the rate of retinal deterioration [65,72] and in some cases even result in long-term survival of retinal cells [73]. Several methods for manipulating individual UPR markers have recently been explored. These include the use of knockout mice, pharmacological agents, and regulation of gene expression via viral vector-mediated gene delivery.

## Transgenic and knockout mice to study the unfolded protein response

Development of knockout mice is important in defining the role of the UPR in the mechanism of retinal degeneration. Several mouse models allow monitoring of the UPR. These include ERAI mice expressing the XBP-1-venus fusion protein [74], ATF6α, ATF6β [75], and IRE [76] knockout mice as well as commercially available ATF4, eIF2a, PERK, caspase-12, caspase-7, and CHOP knockout mice. With the exception of the capsase-12 knockout mice provided by the mutant mouse regional resource center supported by the National Institutes of Health (NIH), the remaining strains are commercially available from Jackson Laboratories.

In our laboratory, we have validated the role of UPR markers including CASP-7, CHOP, and BiP in the progression of adRP using CASP-7, CHOP knockout mice, and overexpression of human BiP cDNA by subretinal adeno-associated viral vector delivery (AAV-BiP/Grp78). These studies have led us to conclude that the rate of retinal degeneration in adRP animals could be modulated by reprograming the UPR. For example, CASP-7 and CHOP are proapoptotic and are activated in adRP animal models [65-67,72]. However, ablations of these two proapoptotic proteins in the T17M RHO retina gave us contrasting results [71,72]. For example, T17M *RHO* CASP-7−/− mice displayed significant preservation of photoreceptors and their function from P30 to P90 compared to the control mice. CASP-7 ablation also protected these mice from a light-induced decrease in ERG responses and apoptosis [72]. Ablation of the proapoptotic CHOP, however, surprisingly expedited retinal degeneration in T17M *RHO* mice [71]. Dark-adapted ERG analysis demonstrated that by 1 month T17M *RHO* CHOP−/− mice already exhibited a 70% reduction in a-wave amplitude compared to T17M *RHO* mice, which was associated with a 22%–24% decrease in the thickness of the outer nuclear layer. Investigating the mechanism of these observed phenomena, we found that the therapeutic effect resulting from ablation of CASP-7 in T17M *RHO* retinas was associated with 1) the reprogramming of the UPR through downregulation of the pATF6 (ATF6 signaling) and ATF4 (PERK signaling) proteins, 2) activation of mammalian target of rapamycin/protein kinase B (mTOR/AKT) by increased production of phosphorylated AKT, and 3) decrease in JNK-induced apoptosis via inhibition of the poly (ADP-ribose) polymerase 1-TNFα-TRAF2-pJNK pathway (Figure 1B). Alternatively, ablation of CHOP in T17M *RHO* retinas upregulated the PERK pathway by increasing levels of peIF2α and diminished IRE1 signaling, thus resulting in decreased spliced Xbp1 (Figure 1C). The findings of these two studies conducted with T17M *RHO* mice suggest that downregulation of the PERK pathway could be a novel therapeutic strategy for treating adRP.

A recent study of AMD that used the conditional RPE knockout of Xbp1 in the mouse retina demonstrated the importance of the XBP1 gene in regulating antioxidant defense in the RPE, and showed that impaired activation of XBP1 may contribute to RPE dysfunction and cell death during retinal degeneration and AMD [61]. The mice in this study expressed less superoxide dismutase 1, superoxide dismutase 2, and catalase in the RPE and experienced increased oxidative stress. The defensive role of XBP1 was also investigated in a study of the *Drosophila* model of retinal degeneration, which found that decreased Xbp1 gene dosage accelerated retinal degeneration in adRP retinas [63].

The role of the ATF4 transcription factor was recently explored with ATF4 knockout mice with diabetic retinas induced by streptozotocin [77]. The study found that loss of ATF4 activity markedly attenuated high glucose-induced production of intercellular adhesion molecule 1, TNF-α, and VEGF. The same study also demonstrated that adenovirus-mediated transduction of ATF4 resulted in elevated intracellular levels of adhesion molecule 1 and VEGF in Müller cells [78], thus suggesting that ATF4 plays a critical role in retinal inflammation signaling and Müller cell–derived inflammatory cytokine production in the diabetic retina. Our laboratory has also validated the role of ATF4 in the activation of ER stress in mouse retinas of OIR (data not shown) [79]. Our findings revealed that the downregulation of ATF4 induced a decrease in VEGF protein and mRNA leading to a subsequent decrease in neovascularization of the hypoxic retina, thus inhibiting disease progression.

## Pharmacological modulation of unfolded protein response markers in animal models of retinal degeneration

The role of individual UPR markers in the progression of human disease was also recently studied with the help of chemical chaperons and pharmacological compounds that augmented either the activity or expression of their target molecules. The first class of modulators include sodium 4-phenylbutyrate (4-PBA) and tauroursodeoxy-cholicacid (TUDCA), which have their origins in traditional Chinese medicine and have been used in humans for various diseases including retinal degeneration [28].

The study of the preservation of retinal ganglia cells (RGCs) by PBA treatment in the rat model of retinal ischemic injury was initiated in 2007 by Jeng et al. [73]. This study demonstrated that nearly 100% of the RGCs were preserved in treated animals compared with controls after preischemic intraperitoneal injection with 100 or 400 mg/kg PBA. Later studies discovered that the chemical chaperone, PBA, rescued the glaucoma phenotypes of Tg-MYOCY437H mice that activate the UPR trabecular meshwork and promote trafficking and secretion of mutant MYOC. This activation is triggered by accumulation of mutant MYOC within the ER [46,80]. An additional study with PBA was conducted in conjunction with the expression of the CNGA3 R563H and Q655X subunits in 661W cells [70]. This study demonstrated that PBA alone with TUDCA had the ability to reprogram the UPR when present in the medium. Under these conditions, spliced Xbp1, BiP, and CHOP mRNAs were significantly reduced suggesting modulation of the ER stress response. Moreover, the maturation and trafficking of localization-defective CNG channels were shown in TUDCA- and PBA-treated cells.

In another study conducted in the rat model of retinal detachment, Mantopoulos et al. concluded that TUDCA treatment does not affect ER stress levels induced by retinal detachment but decreases the level of apoptosis, inhibiting the activity of caspase-3 and -9 and preventing a decrease in the thickness of the outer nuclear layer [81]. The discrepancy between these studies suggests that additional work needs to be done to carefully characterize the therapeutic mechanism of TUDCA.

Commercial availability of pharmacological agents to selectively modulate UPR markers has facilitated the study of individual UPR components and their roles in the progression of retinal degeneration. For example, the role of caspase-12 in mice with Bardet-Biedl Syndrome (BBS), which leads to severe retinal degeneration, was recently described [82]. The study found that the level of apoptosis in BBS−/− mice was reduced from 36% to 26% in response to treatment with the caspase-12 inhibitor. Guanabenz, an inhibitor of the eIF2α phosphatase GADD34, maintained high levels of peIF2α and blocked CAP-dependent translation, thus reducing the apoptosis level to 14%. The authors proposed that the inhibition of CAP-dependent translation in the context of retinal degeneration could prevent deleterious protein overloading in the photoreceptors, thus facilitating their survival. The same study also suggested a more efficient way to maintain photoreceptors by simultaneously increasing BiP activity, inhibiting CAP-dependent translation, and inactivating caspase-12. In our own experimental mouse model (hT17M *RHO* CHOP−/−), a greater than eightfold increase in peIF2a, a translational inhibitor, was observed with accelerated retinal degeneration [71]. Therefore, a decrease in photoreceptor-specific gene expression including mouse and human rhodopsin, as well as cone-rod homeobox and neural retina leucine zipper transcription factors, was unsurprisingly observed together with the accumulation of rhodopsin protein in the outer nuclear layer. Regarding RHO protein, at this point it is not known if it remains within the ER or passes to the Golgi via transport membranes. However, disrupted RHO trafficking and an increase in peIF2α are the hallmarks of accelerated retinal degeneration. The observed increase in peIF2α also demands closer consideration of the details of our experiments. Although this increase is not surprising, it was not originally tested as a primary cause of the expedited retinal degeneration. Therefore, at this point it is not clear whether this increase directly accelerated retinal degeneration in T17M *RHO* mice or was perhaps in response to a potential decrease in GADD34. It is also not clear if the increase in peIF2α demonstrated by Mockel et al. could provide a therapeutic effect in BBS-deficient mice and if such a therapeutic effect would be sustained, since the study was conducted in mouse retinal explants. Therefore, the discrepancy between our study and Mockel et al.’s demands a more careful examination in the future of the role of peIF2.

Another recent study by Chiang et al. [83] demonstrated that selective activation of ATF6 and PERK prevented the cellular accumulation of mutant rhodopsin. These experiments were performed with stable human embryonic kidney 293 (HEK293) cells expressing TetON-ATF6f treated with doxycycline A and with sensitized HEK293 stable cell lines expressing Fv2E-PERK treated with the dimerizing molecule AP20187. The authors concluded that ATF6 signaling may be especially useful in treating retinal degenerative diseases arising from rhodopsin misfolding by preferentially clearing mutant rhodopsin and abnormal rhodopsin aggregates. Another study conducted by the same group also described that selective activation of IRE with 1NM-PP1 in mammalian cells expressing P23H *RHO*, robustly promoted the degradation of misfolded P23H *RHO* via proteasomal and lysosomal degradation pathways without affecting its wild-type counterpart [84]. Together, these studies emphasized that IRE1 and ATF6 signaling may be particularly helpful in preventing chronic ER stress in animal models of retinal degeneration.

The role of GRP78 in the mechanisms of retinal degeneration was also recently examined. The BiP Inducer Protein X (BIX) was tested in mice with tunicamycin or NMDA-induced retinal degeneration [85]. The findings showed that coadministration of BIX (5 nmol) significantly reduced retinal cell death and CHOP expression in RGCs induced by intravitreal injection of tunicamycin or NMDA. Another study of BIX conducted in 661W cells revealed a protective effect of BIX against light-induced cell death [51]. Our group also demonstrated that overexpression of the BiP slowed the rate of retinal degeneration in rats with retinitis pigmentosa [65] (see below). These studies are all in agreement with Chai et al. [86], who proposed that downregulation of GRP78 played a role in the degeneration of trabecular meshwork cells in patients with POAG. This proposal provided molecular insights into the pathogenesis of POAG and suggested that GRP78 may have the potential to be a target for developing new therapies for ER stress–induced trabecular meshwork cell apoptosis.

## Viral gene delivery of unfolded protein response markers

Lentivirus, adenovirus, and adeno-associated vectors (AAV) are effective in delivering genes of interest to the retina and RPE for ocular gene therapy [87]. The use of AAV has become a valuable tool gene therapy. A new variant of this gene transfer system, engineered for ocular diseases, allows effective delivery of genes to the ocular retina after injection into the eye’s easily accessible vitreous humor [88].These latest achievements in AAV-based gene transfer should facilitate the study of molecular mechanisms of pathogenesis due to AAV’s broad cellular tropism, DNA-based genome, lack of known pathogenicity, and broad array of serotypes with unique tissue specificities.

AAV has been used to deliver a wide variety of genes to the retina. For example, the rat diabetic model injected with streptozotocin has been used to validate P58IPK, a 58-kDa inhibitor of protein kinase, as a therapeutic target for diabetic retinopathy. Interestingly, this kinase plays an important role in preventing ER stress [89]. Overexpression of P58IPK was achieved with intravitreal injection of (rAAV2)-P58(IPK), which led to a decrease in the mRNA and protein levels for CHOP, TNFα, and VEGF. This also resulted in a remarkable decrease in vascularization of diabetic retinas demonstrating the protective role of P58IPK.

Our laboratory recently validated the molecular chaperone GRP78/BiP as a therapeutic target for adRP in rat retinas expressing P23H *RHO* (Figure 1D) [65]. We demonstrated that BiP overexpression alleviated ER stress by reducing the levels of cleaved pATF6, peIF2α, and the proapoptotic protein CHOP. We also registered a sustained increase in ERG amplitudes during a 3-month period and later confirmed this result in injected animals monitored over 6 months (data not shown). In addition, we found that the exogenous form of BiP complexed with caspase-12 and the BH3-only protein BiK, which may have contributed to the antiapoptotic activity of BiP. Thus, we concluded that the therapeutic effect provided by overexpressed BiP is due to suppression of apoptosis rather than to the promotion of rhodopsin folding. Similar results have been obtained by Cheetham’s group [90]. The authors revealed that BiP overexpression does not enhance P23H RHO trafficking in SK-N-SHn cells, but is important for maintaining the solubility of rhodopsin in the ER.

## Future perspectives

The rapid increase in the study of ER stress in different models of retinal degeneration has improved our understanding of the mechanisms of retinopathy and highlighted the role of the UPR and individual UPR markers in the pathogenesis of retinal degeneration. This suggests that the UPR markers could be potential therapeutic targets, manipulation of which could alter the rate of retinal degeneration and provide for the survival of retinal neuronal cells. Future work should concentrate on the search for pharmacological molecules that selectively inhibit or induce individual UPR markers. Additionally, an expansion of the list of available UPR animal models will facilitate identifying specific roles for every UPR component in the pathogenesis of retinal degeneration. These approaches, in combination with AAV gene delivery, should be useful tools in manipulating individual UPR markers and performing gene therapy for reprogramming the UPR.
